# Melatonin promotes ripening of grape berry via increasing the levels of ABA, H_2_O_2_, and particularly ethylene

**DOI:** 10.1038/s41438-018-0045-y

**Published:** 2018-08-01

**Authors:** Lili Xu, Qianyu Yue, Guangqing Xiang, Feng’e Bian, Yuxin Yao

**Affiliations:** State Key Laboratory of Crop Biology, Key Laboratory of Biology and Genetic Improvement of Horticultural Crops (Huang-Huai Region, Ministry of Agriculture), College of Horticulture Science and Engineering, Shandong Agricultural University, Tai’an, 271018 China

## Abstract

The role of melatonin in the regulation of fruit ripening and the mechanism involved remain largely unknown. In “Moldova” grape berries, melatonin accumulated rapidly from onset of veraison, reached the maximum at 94 days after bloom (DAB) and then exhibited low levels at late stages of berry ripening. By contrast, abscisic acid (ABA) and hydrogen peroxide (H_2_O_2_) exhibited different accumulation patterns, and ethylene was primarily produced immediately before veraison. Further experiments demonstrated that 10 and particularly 100 µM melatonin treatments increased the levels of ABA, H_2_O_2_, and ethylene production and promoted berry ripening compared with the control treatment, whereas 0.1 and 1.0 µM melatonin did not lead to clear effects. Additionally, the application of inhibitors indicated that ABA, H_2_O_2_, and ethylene participated in the regulation of berry ripening induced by melatonin, and the suppression of ethylene biosynthesis produced the greatest inhibitory effects on melatonin-induced berry ripening compared with those of ABA and H_2_O_2_. Melatonin also promoted ethylene production via ABA. In summary, 10 and particularly 100 µM melatonin treatments promoted berry ripening, which was accomplished, at least partially, via the other signaling molecules of ABA, H_2_O_2_, and particularly ethylene. This research provides insight into melatonin signaling during berry ripening and may advance the application of melatonin to accelerate berry ripening.

## Ripening: waking the grape

Melatonin, the same hormone that regulates sleep and wakefulness in animals, promotes ripening of grapes by triggering the production of other ripening hormones. Melatonin was previously known to play a part in fruit ripening, but the mechanism remains poorly understood. Yu-Xin Yao and co-workers at Shandong Agricultural University in China tested how applying various quantities of melatonin affected ripening of grapes, and also investigated the mechanism of action by measuring levels of other hormones related to ripening, such as ethylene. They found that adding melatonin made grapes ripen sooner, and increasing the quantity increased the effect. Applying substances that inhibited the other ripening hormones repressed ripening, indicating that melatonin cannot act alone. These results illuminate how melatonin triggers ripening, and may help in developing methods to induce ripening in various plants.

## Introduction

Grapevine is one of the most important fruit crops and is planted worldwide. The high economic and nutritional value of grapevine has encouraged many researchers to study the physiological and molecular basis of berry development and particularly berry quality formation. Berry development involves two growth periods; the first period is characterized by rapid cell division and growth and the accumulation of organic acids, and the second period is characterized by the decline in organic acids and the accumulation of sugar, anthocyanin, and flavor compounds. Veraison is a transition phase from the first to the second period^1^.

Grapes are classified as non-climacteric fleshy fruits and might be considered a model species to study the ripening of non-climacteric species^2^. Although the mechanism involved in the ripening of non-climacteric fruits remains largely unclear, several signaling molecules participate in the control of ripening in grape berry^3,4^. Based on the substantial accumulation during fruit maturation in non-climacteric fruits, abscisic acid (ABA) plays an important role in accelerating fruit ripening, and based on RNA-Seq analysis, is a primary regulator of grape berry ripening onset^5,6^. Additionally, one of the best-known roles of ABA is the ability to upregulate anthocyanin production of grape berries^7^. By contrast, a few recent studies show that grape berry tissues have a fully functional pathway for ethylene biosynthesis that is activated immediately before veraison; furthermore, ethylene perception is critical for some grape berry ripening^8,9^. Exogenous ethylene also positively affects anthocyanin production in grape berry^8,10^. Additionally, reactive oxygen species (ROS) are involved in regulating fruit ripening and senescence^11^, and H_2_O_2_ can regulate the process of ripening by regulating ripening-related genes^12^. Particularly, the accumulation of ROS is a characteristic of grape berry ripening^13^, and H_2_O_2_ promotes the early ripening of “Kyoho” berry^4^.

Melatonin (*N*-acetyl-5-methoxytryptamine) is an indoleamine that is synthesized from l-tryptophan metabolism via serotonin. Some recent studies report that melatonin participates in the regulation of fruit ripening and senescence. Melatonin promotes ripening and improves quality of climacteric tomato fruit during postharvest life^14^, and further proteomics analysis provides insights into the physiological and molecular mechanisms underlying melatonin-mediated tomato fruit ripening^15^. In banana, another typical climacteric fruit, melatonin is also an indicator for fruit ripening in various varieties^16^. By contrast, exogenous application of melatonin delays postharvest senescence in banana, strawberry, and peach fruits^16–18^. Therefore, melatonin treatment is proposed to positively regulate fruit ripening but negatively regulate fruit senescence, although the two processes are intrinsically linked^15,16^. However, melatonin also regulates fruit metabolism or abiotic resistance via interaction with other signaling molecules. Melatonin treatment increases ethylene production and accelerates the climacteric phase of tomato fruits^14^, but exogenous application of melatonin reduces ethylene production of banana through regulation of the expression of *ACO1* and *ACS1*^16^. Additionally, melatonin treatment can alter ABA levels, e.g., melatonin treatment results in a rapid decrease in ABA levels during seed germination of cucumber under salt stress and in apple leaves in drought conditions through regulation of ABA biosynthesis and catabolic enzymes^19,20^. By contrast, the application of melatonin results in increased ABA content in drought-primed plants when exposed to cold stress^21^. Moreover, melatonin delays senescence of peach fruit partly via decreasing the levels of superoxide anion and H_2_O_2_^17^.

The objectives of this study were to investigate the changes in melatonin and the other signaling molecules, including ABA, H_2_O_2_, and ethylene during berry ripening, to elucidate the effects of exogenous melatonin application on berry ripening and the accumulations of the three signaling molecules, and to reveal whether melatonin can regulate berry ripening through the other signaling molecules. The underlying mechanism of melatonin in the regulation of berry ripening was further revealed in this study, and these results may help promote the application of melatonin in regulating berry ripening.

## Results

### Changes in total soluble solids, titratable acid and anthocyanin during berry ripening

**Fig. 1:**
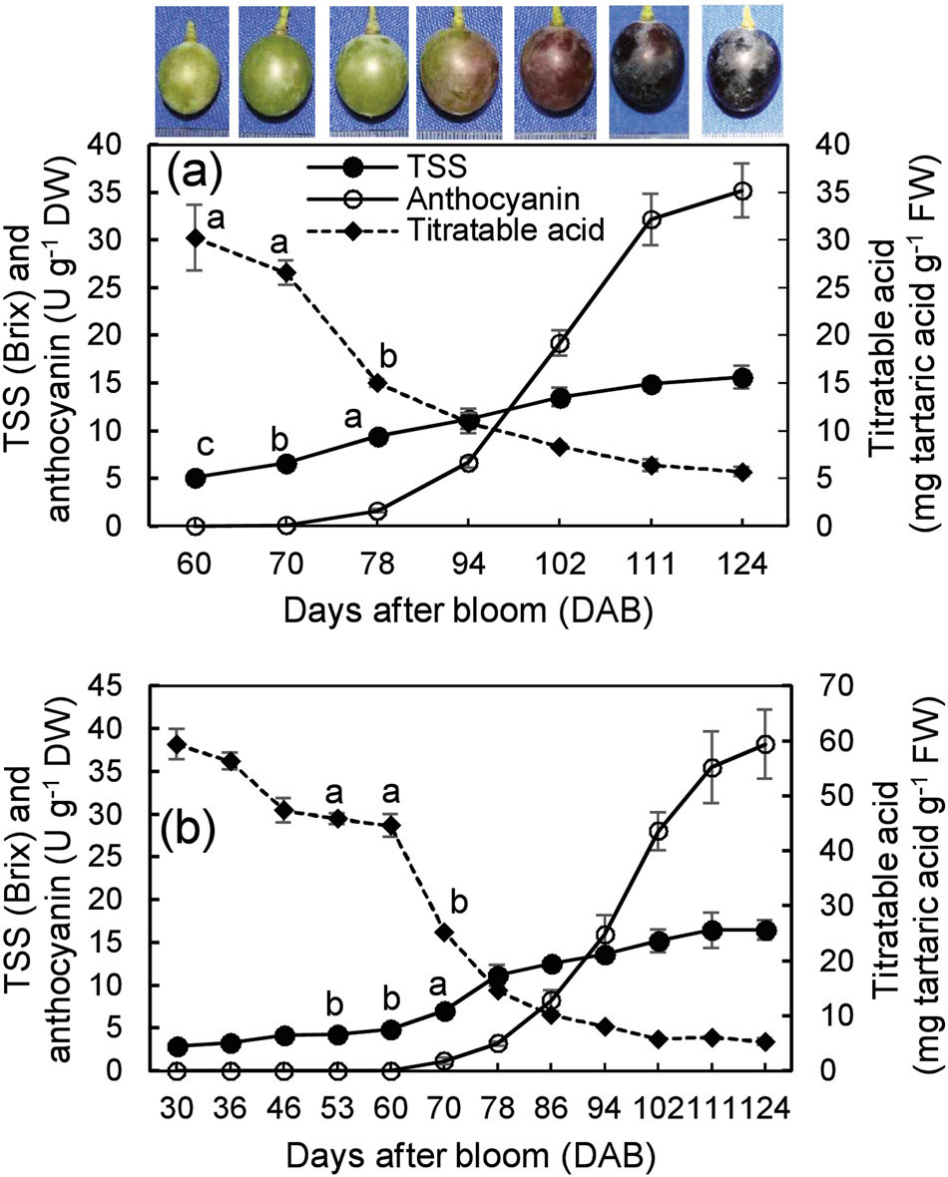
Berry coloration and changes in berry TSS and anthocyanin and titratable acid. The data in panels **a** and **b** were determined in 2016 and 2017, respectively. DW dry weight, FW fresh weight. Values represent the mean ± SD of three replicates, and each replication contained 240 berries, which were randomly sampled from the middle of the clusters of 10 vines. The differences are not significant at the 5% significance level among the values with the same small letter at the different time points

The onset of ripening (veraison) is indicated by increases in sugar content, and in red and black grapes, anthocyanin biosynthesis and by decline in acid content. In 2016, titratable acid continued to decrease and total soluble solids (TSS) continued to increase from 60 days after bloom (DAB), anthocyanin began to accumulate at 78 DAB, and coloring began at 94 DAB (Fig. 1a). In 2017, particularly with more sampling time points, the dramatic decrease in titratable acid and TSS beginning to continuously and prominently accumulate from 60 DAB were more clear than in 2016; and anthocyanin was detectable at 70 DAB, followed by the rapid increase (Fig. 1b). Titratable acid reached the minimum, and the TSS and anthocyanin contents reached their maximums in the ripened berries (124 DAB) (Fig. 1a, b). Therefore, the onset of veraison occurred approximately at 70 DAB, which varied slightly between the two years.

### Changes in 5-hydroxyserotonin, melatonin and 2-hydroxymelatonin, and the other signaling molecules during berry ripening

**Fig. 2:**
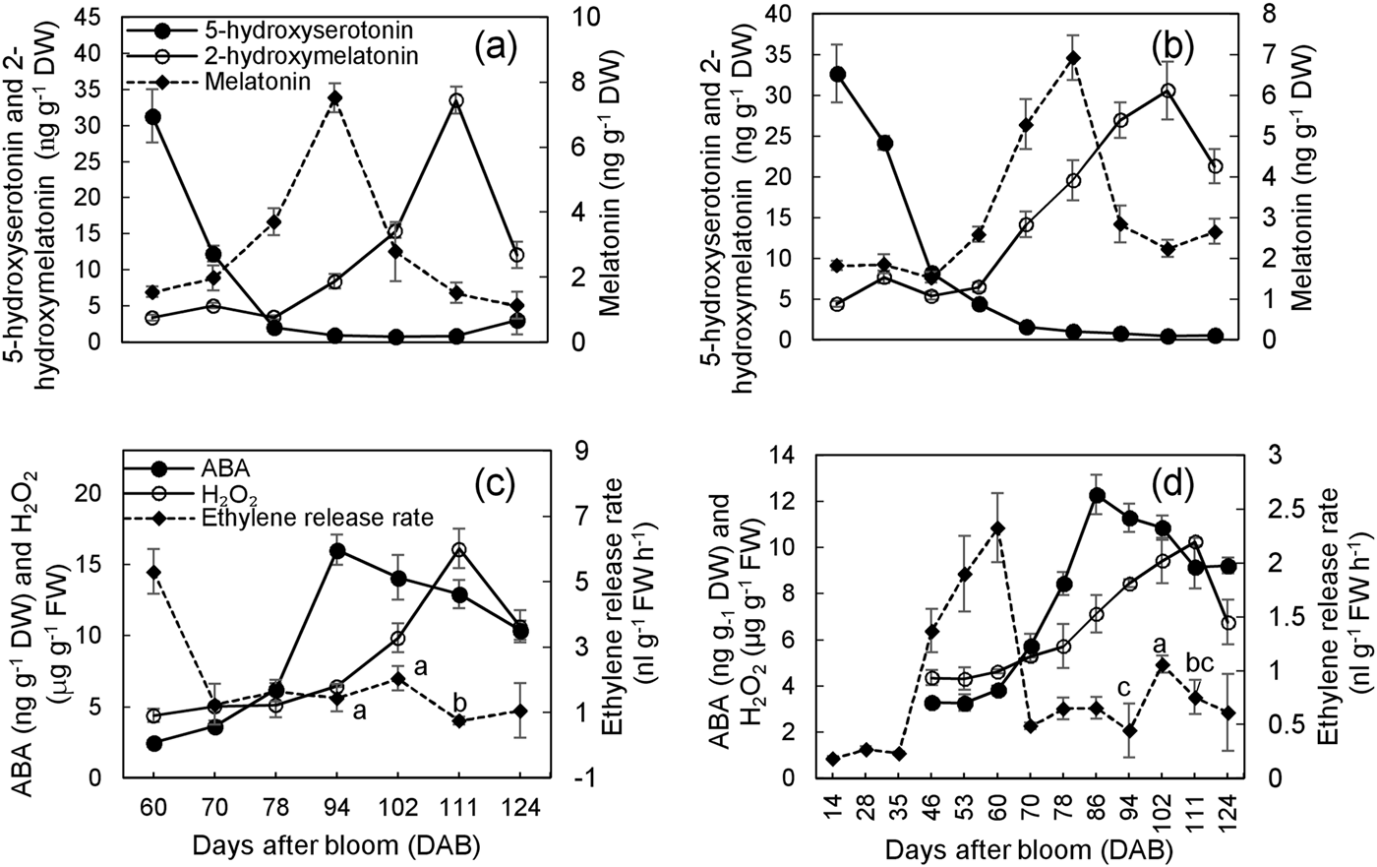
Accumulations of melatonin and its precursor (5-hydroxyserotonin) and metabolite (2-hydroxymelatonin) and the other signaling molecules during berry ripening. The data in (**a**) and (**b**) were determined in 2016, and those in (**c**) and (**d**) were from 2017. In (**d**), ethylene release rate was detected from 14 DAB, and ABA and H_2_O_2_ were detected from 46 DAB. DW dry weight, FW fresh weight. Values represent the mean ± SD of three replicates, and each replication contained 240 berries, which were randomly sampled from the middle of the clusters of 10 vines. The differences are not significant at the 5% significance level among the values with the same small letter in (**c**) and (**d**)

When veraison occurred, melatonin increased rapidly and peaked at 94 DAB with a 3.89- and 3.54-fold increase compared with melatonin at 70 DAB in 2016 and 2017, respectively (Fig. 2a, b). Thereafter, melatonin declined sharply at 102 DAB, but different changes were found at 111 and 124 DAB between the two years (Fig. 2a, b), suggesting the influence of environmental factors. By contrast, 2-hydroxymelatonin also promptly accumulated during berry ripening but reached the maximum at 111 DAB. Additionally, 5-hydroxyserotonin, a precursor of melatonin biosynthesis, decreased sharply from the first sampling time point and remained at relatively low levels in the late stages (Fig. 2a, b), indicating the transformation of 5-hydroxyserotonin into melatonin.

ABA continuously accumulated from 60 DAB and reached the peak at 94 DAB, which was 2.82-fold higher than that at 60 DAB in 2016 (Fig. 2c). By contrast, the determinations in a small time interval showed that the peak of ABA occurred at 86 DAB in 2017 (Fig. 2d). Thereafter, ABA content declined slowly but remained at a high level (Fig. 2c, d). By contrast, the peak of H_2_O_2_ was at 111 DAB in both years, and the concentration of H_2_O_2_ at the peak was 1.42- and 1.21-fold higher than the value at 60 DAB in 2016 and 2017, respectively (Fig. 2c, d). In 2016, high ethylene release rates were detected at 60 DAB, with relatively low values found at the other time points, suggesting an ethylene peak at 60 DAB or earlier time point (Fig. 2c). Further determinations in 2017 indicated that the peak of ethylene release also occurred at 60 DAB, which was 3.80-fold higher than that at 70 DAB (Fig. 2d). Additionally, a small peak of ethylene release was observed at 102 DAB in 2016 and particularly in 2017 (Fig. 2c, d)

Collectively, the peak of ethylene release occurred before veraison, and the peaks of ABA, melatonin, and 2-hydroxymelatonin/H_2_O_2_ occurred in chronological order after onset of veraison.

### Exogenous melatonin treatments increased the levels of melatonin and the other signaling molecules and promoted berry ripening

**Fig. 3:**
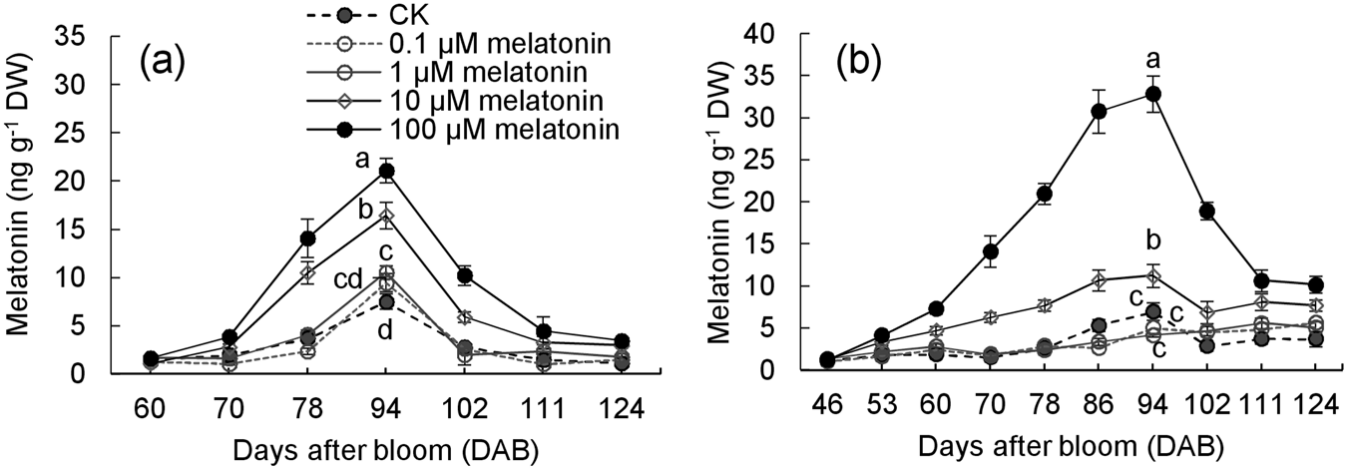
**Changes in melatonin content of grape berries under the treatments of different concentrations of exogenous melatonin.** The berries were treated once at 60 DAB in 2016 (**a**) and were treated two times at 46 and 53 DAB in 2017 (**b**). DW dry weight. Values represent the mean ± SD of three replicates, and each replication contained 240 berries, which were randomly sampled from the middle of the clusters of 10 vines. The differences are not significant at the 5% significance level among the values with the same small letter at the same DAB

The role of melatonin in promoting berry ripening was evaluated by applying exogenous melatonin treatment. First, the influences of exogenous melatonin concentration on the content of berry melatonin were determined (Fig. 3). In 2016, the content of melatonin in berries treated once with 10- and 100-µM melatonin at 60 DAB largely increased, and the increments increased with the increase in concentration of applied melatonin. Increments of 1.18- and 1.80-fold were generated at 94 DAB in the 10- and 100-µM melatonin-treated berries, respectively, compared with the control check (CK) berries. By contrast, 0.1- and 1.0-µM melatonin treatments did not lead to clear changes in melatonin content of berries (Fig. 3a). To further increase melatonin levels before the occurrence of the ethylene release peak, melatonin treatments at two times, i.e., at 46 and 53 DAB, were performed in 2017 (Fig. 3b). Two-time treatment with 10- and 100-µM melatonin largely increased melatonin levels of grape berries and led to 3.89- and 2.51-fold increments, respectively, at 60 DAB compared with the level in the CK treatment. The increase reached 1.61- and 4.74-fold at 94 DAB under 10- and 100-µM melatonin treatments, respectively, compared with the CK (Fig. 3b). By contrast, 0.1- and 1.0-µM melatonin treatment did not clearly alter melatonin levels, similar to results in 2016 (Fig. 3a, b).

**Fig. 4:**
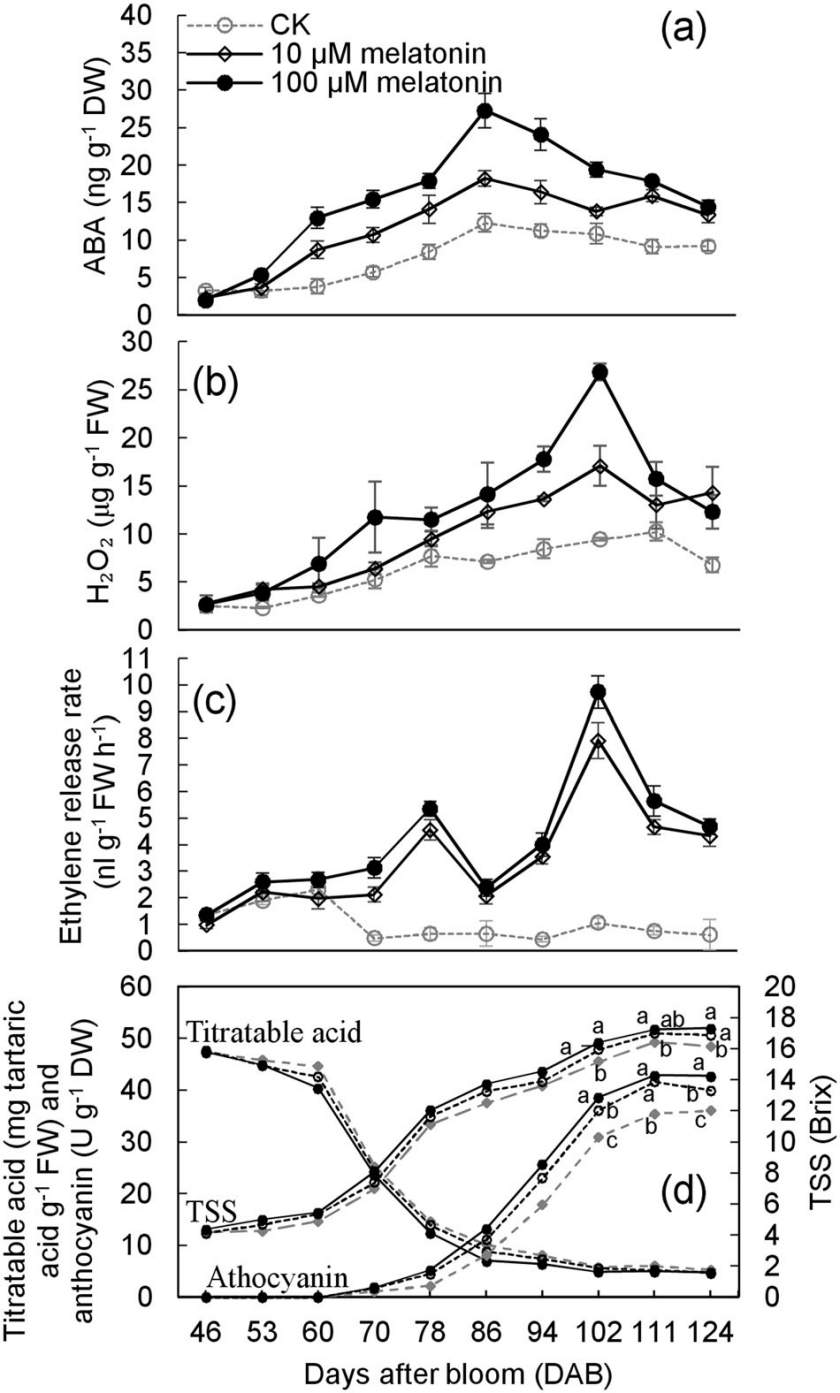
Changes of the signaling molecules and the ripening-related parameters under melatonin treatments. ABA (**a**), H_2_O_2_ (**b**), ethylene release rate (**c**) and the ripening-related parameters (**d**) were determined in 2017. The berries were treated two times at 46 and 53 DAB. The ripening-related parameters included TSS, anthocyanin, and titratable acid (**d**). FW fresh weight, DW dry weight. Values represent the mean ± SD of three replicates, and each replication contained 240 berries, which were randomly sampled from the middle of the clusters of 10 vines. The differences are not significant at the 5% significance level among the values with the same small letter at the same DAB in (**d**)

Second, the effects of the 10- and 100-µM melatonin treatments on ABA, H_2_O_2_, and ethylene release were determined in 2017. Treated with 10-µM melatonin, berries accumulated 30.0% more ABA than that of the control berries from 60 to 124 DAB, and treatment with 100-µM melatonin caused greater increases in ABA content than did 10-µM melatonin (Fig. 4a). Similarly, exogenous melatonin treatments promoted the accumulation of H_2_O_2_ at the same time points, and 100-µM melatonin caused a greater effect on H_2_O_2_ than that of 10-µM melatonin. Moreover, with melatonin treatments, the peak of H_2_O_2_ occurred in advance (Fig. 4b). By contrast, melatonin treatments not only largely increased ethylene release rate of berries but also altered accumulation patterns (Fig. 4c). In the CK berries, the ethylene release rate decreased from 70 DAB and then remained at very low levels. By contrast, two peaks of ethylene release were produced at 78 and 102 DAB in the melatonin-treated berries. At 78 DAB, 7.28- and 6.06-fold increments in melatonin content were observed in the 100- and 10-µM-treated berries, respectively, compared with the control berries, and more than 11-fold increments were produced under the 100- and 10-µM treatments at 102 DAB (Fig. 4c).

Last, the effects of 10- and 100-µM melatonin treatments on berry ripening were determined in 2017 (Fig. 4d). The berries treated with melatonin at 10 and particularly 100 µM accumulated high TSS and anthocyanin and low titratable acid compared with those of the CK berries at the same time points. Additionally, contents of TSS and anthocyanin in the melatonin-treated berries at 102 DAB surpassed the values in the ripened CK berries, and the melatonin-treated berries at 102 DAB accumulated lower titratable acid than that in the ripened CK berries. Therefore, 10- and 100-µM melatonin treatments promoted berry ripening.

In summary, exogenous melatonin treatments increased the levels of melatonin, ABA, H_2_O_2_, and ethylene production in the berries and promoted berry ripening, suggesting that melatonin might promote berry ripening via these three signaling molecules.

### Exogenous melatonin treatments promoted berry ripening via the signaling molecules ABA, ethylene, and H_2_O_2_

**Fig. 5:**
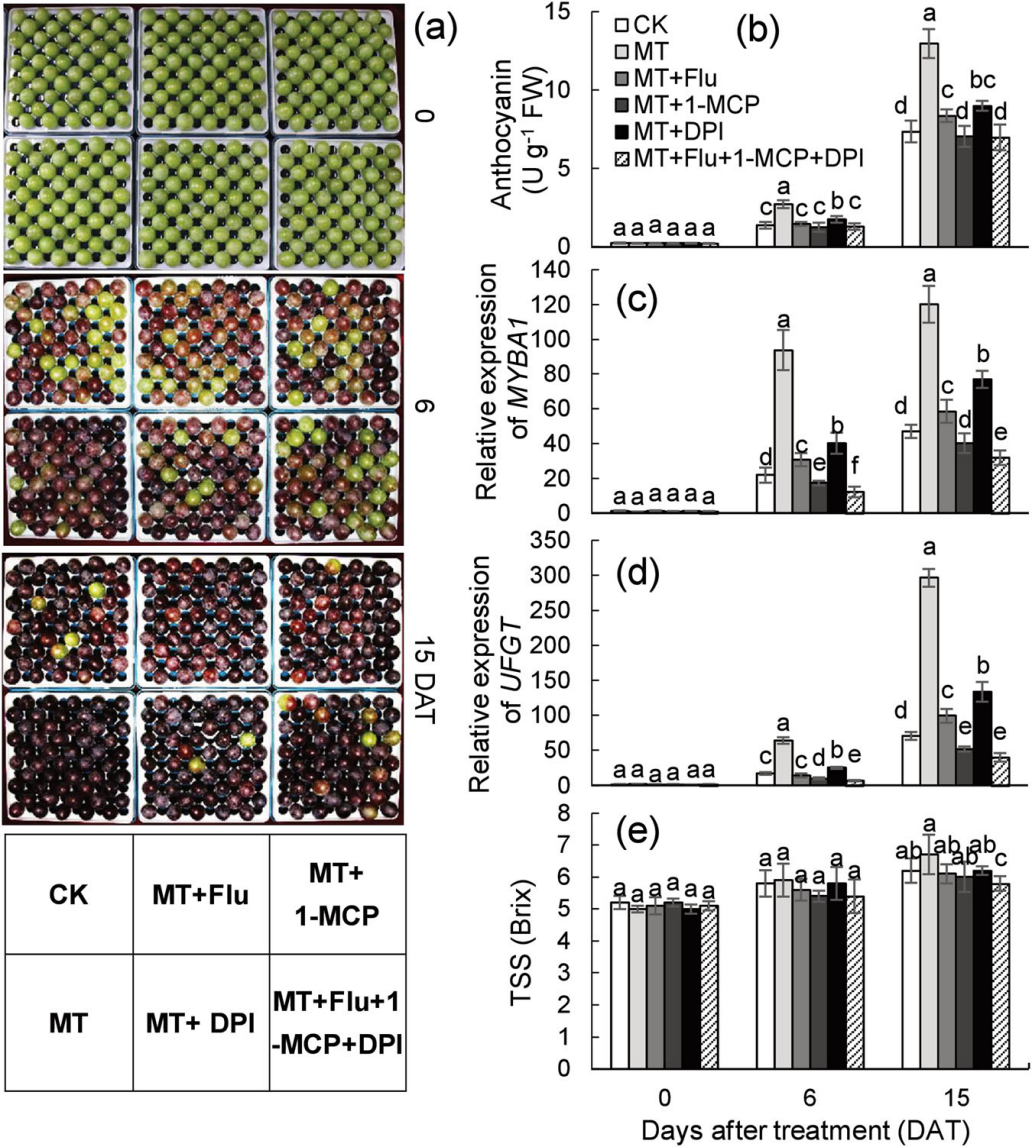
**Changes in the ripening-related parameters and expression of** ***MYBA1*** **and** ***UFGT*** **in the berries with different treatments.** The berries at 60 DAB were used for different treatments. Half of each berry cluster was immersed in the treatment solution, which was refreshed every 3 days. The photos (**a**) were taken and the corresponding anthocyanin content (**b**), gene expression (**c**, **d**) and TSS accumulation were determined at different DAT. MT melatonin, Flu fluridone, DPI diphenylene iodonium, 1-MCP 1-methylcyclopropene. Values represent the mean ± SD of three replicates, and each replication contained 50 berries, which were randomly sampled from the same vine. The differences are not significant at the 5% significance level among the values with the same small letter at the same DAT in (**b**–**e**)

To further investigate whether melatonin promoted berry ripening via the other signaling molecules, melatonin, fluridone (Flu; an inhibitor of ABA biosynthesis), diphenylene iodonium (DPI; an inhibitor of H_2_O_2_ biosynthesis), and 1-methylcyclopropene (1-MCP; an inhibitor of ethylene response at the receptor level) were used to treat the berries in vitro at 60 DAB in 2017 (Fig. 5). When the berries were treated with melatonin at 100 µM, most accumulated much anthocyanin and exhibited red purple color at 6 days after treatment (DAT), in contrast to only approximately 65% of the CK berries (Fig. 5a). A 1.9- and 1.76-fold increase of anthocyanin in melatonin-treated berries was also detected at 6 and 15 DAT, respectively, compared with the CK berries (Fig. 5b). By contrast, the treatment of melatonin plus DPI, Flu, or 1-MCP significantly reduced the anthocyanin content at 6 and 15 DAT compared with the treatment of melatonin alone. The application of 1-MCP caused the greatest inhibitory effects on melatonin-induced anthocyanin accumulation, and the berries under melatonin plus 1-MCP treatment accumulated similar levels of anthocyanin to those of the CK berries. By contrast, the anthocyanin content in melatonin plus Flu or DPI was significantly higher than that in the CK berries. The treatment of melatonin plus all three inhibitors produced similar effects on anthocyanin to those of the treatment of melatonin plus 1-MCP (Fig. 5a, b). Additionally, the gene expression of *MYBA1* and *UFGT*, two marker genes in the regulation of anthocyanin biosynthesis, indicated the same results as those of anthocyanin assays (Fig. 5c, d). For TSS, the patterns of change were similar to those of anthocyanin under the different treatments at 15 DAT, although the differences were small, and the treatment of melatonin plus the three inhibitors significantly reduced the melatonin-induced increase in TSS (Fig. 5e). Therefore, exogenous melatonin treatments promoted berry ripening, at least partially, via ethylene, ABA, and H_2_O_2_, and ethylene produced the greatest effect on ripening compared with that of ABA and H_2_O_2_ under the applied conditions.

### Melatonin promoted ethylene release partially via ABA

**Fig. 6:**
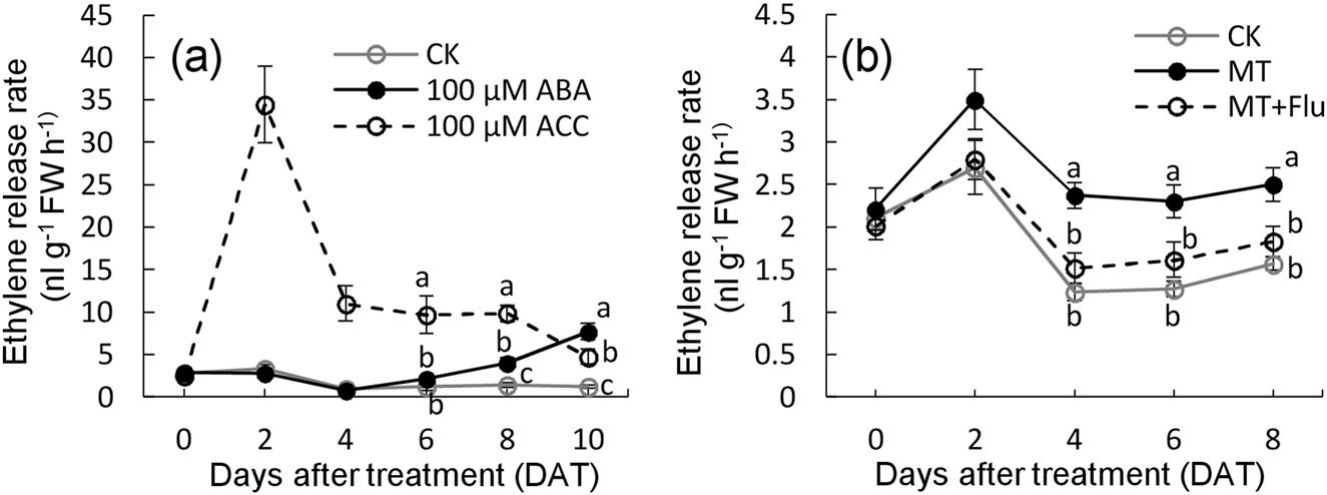
Modifications in ethylene release rate in the berries with different treatments. The berries at 60 DAB were used in the in vitro treatment, i.e., half of each berry cluster was immersed in the treatment solution, which was refreshed every 3 days. The berries at different DAT were used for the determinations of ethylene release rate. The solution of 100 µM 1-aminocyclopropane-1-carboxylate (ACC) and water (CK) were used as positive and negative controls, respectively. MT melatonin, Flu fluridone, FW fresh weight. Values represent the mean ± SD of three replicates, and each replication contained 50 berries, which were randomly sampled from the same vine. The differences are not significant at the 5% significance level among the values with the same small letter at the same DAT

In a previous study, ABA treatment promoted ethylene release in grape pulp^22^, and therefore, we determined whether melatonin could promote ethylene release by increasing levels of ABA (Fig. 6). Under CK conditions, ethylene release in berries declined after a small increase and then remained at a low level. Ethylene release rate largely increased with 1-aminocyclopropane-1-carboxylate (ACC) treatment (as a positive control) at 2 DAT and thereafter decreased gradually. By contrast, ABA treatment promoted ethylene production from 6 DAT and led to a higher level of ethylene production than that with ACC at 10 DAT (Fig. 6a). Additionally, ethylene release rate was determined under the treatments of MT and MT + Flu (Fig. 6b). The results showed that melatonin promoted ethylene production of the in vitro berries, but the application of Flu largely reduced the increase in ethylene production caused by melatonin treatment. For example, melatonin treatment produced a 1.59-fold increase in ethylene release rate compared with that of the CK, whereas the increment declined to 1.17-fold under the treatment of MT + Flu at 8 DAT. Collectively, these results indicated that melatonin promoted ethylene biosynthesis partially via ABA.

## Discussion

### Melatonin plays different roles in the regulation of berry ripening and senescence

The accelerated accumulations of TSS and anthocyanins and the declines in titratable acid were indications of the role of melatonin in promoting berry ripening. Additionally, the role of melatonin in the regulation of ripening was dose-dependent (Fig. 4d). Similar increases in TSS, glucose, and pigment accumulations are also observed in melatonin-treated tomato fruits^14^. However, melatonin treatment decreases total soluble sugars or solids and delays postharvest senescence of peach and banana fruits, and a concentration-dependent effect is observed in banana fruits^16,17^. In particular, exogenous application of melatonin positively regulates fruit ripening; whereas, fruit senescence is negatively regulated^15^. Therefore, melatonin may play different roles in regulating berry ripening and senescence, and the different roles might correspond to two functions of melatonin. First, melatonin can act as an endogenous elicitor and signaling molecule^23^. One of the first roles proposed for melatonin in plants was possible action as a growth regulator because of the structural similarity to indole-3-acetic acid (IAA);^23^ some studies also indicate that melatonin can promote plant growth with a considerable auxinic effect^24^. Additionally, melatonin promotes seed germination in cucumber^19^, promotes tomato ripening^14^, and increases the size and synchronicity of grape berries^25^. Moreover, the multiple changes in gene expression caused by melatonin are an indication of a role as a multiregulatory molecule capable of coordinating many aspects of plant development^23^. Therefore, melatonin may promote fruit ripening as a signaling molecule. Second, melatonin, as a “suicide” antioxidant, can directly scavenge radicals and radical products^23^. Fruit senescence is related to reactive oxygen metabolism and membrane lipid peroxidation, which can directly lead to tissue death^26^. Melatonin decreases the levels of superoxide anion and H_2_O_2_ and delays fruit senescence of peach^17^ and also delays physiological deterioration of cassava roots postharvest by lowering H_2_O_2_ accumulation^27^. Therefore, melatonin might delay fruit senescence via scavenging ROS as an antioxidant.

### Roles of melatonin, ABA, H_2_O_2_, and ethylene in triggering or promoting berry ripening

Many studies indicate that the patterns of melatonin accumulation during ripening are different among fruit species, and that the mechanism of melatonin to regulate fruit ripening is complicated. Melatonin levels continue to decrease with fruit ripening in mulberry;^28^ however, the highest melatonin content is detected in the ripest tomato fruit and sweet cherries^29,30^. In the “Moldova” berry, melatonin exhibited a peak in the berries at 94 DAB in late veraison (Fig. 2a, b); similarly, melatonin content at veraison is higher than that at preveraison in seed and flesh of “Merlot” grape^31^. Additionally, the continuous decline in 5-hydroxyserotonin indicated transformation into melatonin at veraison (Fig. 2a, b). Melatonin can be directly transformed to 2-hydroxymelatonin by the catalysis of melatonin 2-hydroxylase^32^. Comparison of the changing patterns in melatonin and 2-hydroxymelatonin suggested the transformation of melatonin into 2-hydroxymelatonin after veraison under the condition of low supply of 5-hydroxyserotonin (Fig. 2a, b). Therefore, melatonin might be primarily accumulated at veraison in grape. Comparisons of melatonin level and the parameters involved in ripening showed that the abrupt increase in melatonin content coincided with the increases in TSS and total anthocyanin content at veraison, but anthocyanin and TSS continued to increase throughout the ripening period, whereas simultaneously, melatonin levels started to decrease (Figs. 1a, b and  2a, b)^33^. All these results suggest that melatonin is a regulator of berry ripening onset.

Numerous studies of ABA determination, transcriptomics, and proteomics show that ABA plays a primary role in controlling the berry ripening process^3,6^. Although sugar accumulation correlates with an increase in ABA content of the berry^33^, ABA treatment does not affect sugar content, and ABA treatment at 3 weeks before veraison even decreases the sugar content of the berry^33,34^. Additionally, ABA did not necessarily sustain the accumulation of anthocyanin and TSS, because anthocyanins and TSS continued to increase throughout the ripening period, whereas ABA started to decrease from 94 DAB (Figs. 1a, b and 2c, d)^33^. Therefore, ABA might also be a regulator of berry ripening.

Although grape berry is a non-climacteric fruit, an oxidative burst occurs with a characteristic rapid accumulation of H_2_O_2_^13^, and the H_2_O_2_ likely acts as a signaling molecule to accelerate berry ripening of grape rather than as a toxic by-product in the ripening of the berry^4^. Strong evidence also indicates the involvement of H_2_O_2_ in tomato ripening^12^. In this study, H_2_O_2_ rapidly accumulated from 86 DAB and peaked at 111 DAB (Fig. 2c, d), suggesting that the role of H_2_O_2_ in regulating berry ripening occurred after the initiation of berry ripening. H_2_O_2_ has been reported as a potential signaling molecule that regulates fruit ripening in the middle stage of fruit development but may also function as a primary toxic molecule in the late stage of peach fruit^35^. These studies show that the role of H_2_O_2_ as a signal or toxic molecule depends on the fruit developmental stage.

During grape ripening, the typical respiration peak does not occur. Nevertheless, the peak of ethylene release occurred immediately before veraison in “Moldova” grape (Fig. 2c, d); similar results are also reported in the grape cultivar “Muscat Hamburg”^22^ and Cabernet Sauvignon^8^. Additionally, the peak of ACC oxidase transcript accumulation also occurs immediately before veraison or in green berries in other grapevine cultivars^36,37^. These results all provide support that the peak of ethylene release occurs before veraison. Therefore, ethylene might be involved in the trigger of berry ripening. Additionally, the application of 2-chloroethylphosphonic acid (2-CEPA), an ethylene-releasing compound, on berries at veraison promotes anthocyanin accumulation in Cabernet Sauvignon berries;^38^ however, 2-CEPA application delayed ripening when applied early in berry development^39^, which might involve an increase in production of IAA caused by ethylene^40^. From the above discussion, the inference is that ethylene might regulate berry ripening via interplay with other regulators, whereas the effects of exogenous ethylene on berry ripening depend on the developmental stage of the berries at the time of application.

Collectively, melatonin, ABA, and H_2_O_2_ can promote berry ripening; however, their exact role as either a primary promoter or simply a participant in controlling berry ripening requires further research. Ethylene is a potential signaling molecule that can trigger berry ripening, but more decisive evidence is required.

### Melatonin promotes berry ripening via other signaling molecules

Grape berry development and ripening is a complex process, and berry ripening involves the integration of multiple hormonal signals, with some hormones acting as promoters and others as repressors. The mechanisms involved in perceiving and signaling melatonin during berry ripening remain poorly understood. In order to reveal the interaction of melatonin with other signaling molecules in berry ripening, exogenous melatonin treatment was performed and the biosynthesis of ABA and H_2_O_2_ and ethylene signaling were inhibited by applying their specific inhibitors, Flu, DPI, and 1-MCP^22,41^.

In this study, melatonin treatment increased the ABA levels of grape berries, and ABA mediated the melatonin-induced berry ripening (Figs. 4a and 5). Some published data indicate that ABA might act as a downstream signal of melatonin in a stress response, because neither ABA nor fluridone changes endogenous melatonin^42^. Additionally, some studies indicate a role for melatonin in regulating ABA levels, but different effects are observed. For example, melatonin treatment increases ABA accumulation and alleviates cold-induced oxidative damage in leaves of *Elymus nutans*^42^, but melatonin decreases ABA levels during seed germination under salt stress and in heat-induced senescence^19,43^. Therefore, melatonin can regulate ABA metabolism and signaling but causes varying effects under different conditions or in different tissues.

The effects of melatonin behavior on ethylene are complicated. Results indicated that ethylene mediated melatonin-induced berry ripening (Fig. 5); similarly, melatonin treatment promotes ethylene biosynthesis in tomato postharvest ripening^14^. However, in banana postharvest ripening, melatonin treatment reduces ethylene production through regulation of the expression of *ACO1* and *ACS1*^16^. These opposite effects of melatonin on ethylene suggest that the regulation of melatonin on ethylene might involve the other regulators of ethylene biosynthesis. This hypothesis is supported by a study in which melatonin treatment inhibits ethylene production in banana leaves, whereas combined treatments of melatonin and *Fusarium* wilt cause induction of ethylene levels^44^. Additionally, several studies reveal that ABA treatment can induce ethylene evolution in fruit^22,45^. In particular, this study determined that ABA induced ethylene production and mediated melatonin-induced ethylene release (Fig. 6). Therefore, melatonin promoted ethylene production at least partially via ABA in berry ripening. This conclusion could also explain the peak in ethylene production at 102 DAB, which might be attributed to the high level of ABA under melatonin treatment.

This study revealed that melatonin increased the levels of H_2_O_2_ during berry ripening, and that H_2_O_2_ participated in melatonin-induced berry ripening (Figs. 4b and 5). Melatonin and H_2_O_2_ as antioxidant and ROS, respectively, did not exhibit an adversarial relationship, suggesting that melatonin and H_2_O_2_ act predominantly as signaling molecules during berry ripening. By contrast, melatonin scavenges ROS as an antioxidant in fruit senescence of banana and peach^16,17^. The interplay between melatonin and H_2_O_2_ remains largely unknown and awaits further studies.

## Conclusions

Melatonin and ABA prominently accumulated at veraison, and thereafter, although melatonin sharply declined, ABA remained at relatively high levels; the large accumulation of H_2_O_2_ was late compared with that of melatonin and ABA during berry ripening. By contrast, the strongest ethylene production occurred before veraison. The different accumulation patterns indicated their different roles in the regulation of berry ripening. Additionally, 10- and 100-µM melatonin treatments increased the levels of ABA, H_2_O_2_, and ethylene production, and promoted berry ripening in a concentration-dependent manner by increasing endogenous melatonin content. Further experiments determined that ABA, H_2_O_2_, and particularly ethylene participated in melatonin-induced berry ripening. Moreover, melatonin promotion of ethylene production was partially dependent on ABA. In summary, melatonin promoted berry ripening at least partially through ABA, H_2_O_2_, and particularly ethylene.

## Materials and methods

### Plant materials and experimental treatments

The present experiment was undertaken at an experimental vineyard in Tai-An City (36°.17′N, 117°.16′E), Shandong Province, China. Daily average temperature in the experimental site ranged between 7.5 and 31.0 °C from April to August (Fig. S1). The grapevines used in this study were “Moldova” (*Vitis vinifera* × *labrusca*). The vines, each of which had 15 vertical fruiting shoots on the horizontal cordon, were planted at a row × vine spacing of 2.5 × 2.0 m. Each fruiting shoot was controlled to produce two clusters. In 2016, the berries at 60 DAB were treated with melatonin. Each grape cluster on a vine was soaked for 5 s in melatonin solution at a concentration of 0.1, 1.0, 10.0, or 100 µM plus 0.05% (v/v) Triton X-100 in a 2000-mL plastic beaker. CK berries were soaked in 0.05% (v/v) Triton X-100. In 2017, melatonin treatments were applied twice at 46 and 53 DAB using the same methods. Additionally, the berries at 60 DAB were collected for in vitro treatment in 2017. The berries were soaked in solutions of 100-µM melatonin plus 50-µM Flu, 200-µM DPI, or 5 µL L^−1^ 1-MCP under a 14-/10-h (light/dark) photoperiod at approximately 600 mmol m^−2^ s^−1^ at 25 °C for 15 days. The berries at different DAB or DAT were collected, rinsed, frozen in liquid nitrogen, and stored at −70 °C for the determinations of ripening-related parameters, melatonin, and other hormones. Each treatment was conducted with three replications, and each replication contained 10 vines and 50 berries for in vivo and in vitro treatments, respectively.

### Determination of relative anthocyanin content, TSS, and titratable acid

Relative anthocyanin content was determined according to the method described by Neff and Chory^46^ with minor modifications. Ten milliliters of methanol in a 1% (v/v) HCl solution was added to 0.5 g ground lyophilized berry skins and 1.5 g fresh berry skins from in vivo and in vitro treatments, respectively. Sonicated for 3 min. After 24 h of incubation in the dark, the extraction was centrifuged for 10 min at 10 000 rpm, and the aqueous phase was subjected to spectrophotometric quantification at 530 and 657 nm. The relative unit was calculated by the formula OD = A530 − 0.25 × A657. The relative anthocyanin content is expressed as U mg^−1^ dry weight or fresh weight (FW).

Fresh berry pulp was mixed, homogenized, and filtered. The filtrate was used for the determination of TSS and titratable acid. TSS was measured using a manual, digital-displayed sugar meter (PAL-1; Atago, Tokyo, Japan), and the results are expressed as Brix. Titratable acid was determined by the titration of the filtrate with 0.1 M NaOH to an end point at pH 8.3. The results are expressed as mg tartaric acid per g FW.

### Extraction and determination of 5-hydroxyserotonin, melatonin, and 2-hydroxymelatonin

Melatonin was extracted as previously reported^14^ with some modifications. The extraction was conducted entirely under dim green light. One gram of ground lyophilized grape (skin and pulp) was extracted three times in 8 mL of methanol via an ultrasonic bath for 15 min each time. After centrifuging at 5000 rpm for 15 min, the supernatant was filtered on a filter paper, and the filtrate was evaporated to dryness at 30 °C in a rotary evaporator. The residue was dissolved in 5 mL of chromatographic grade methanol and transferred to a C_18_ solid phase extraction cartridge (ProElut^TM^; DIKMA, China) for the purification of melatonin.

The samples were separated on an Acquity UHPLC system (Waters, Milford, MA, USA). The separation was performed by injecting 10 µL of sample onto a BEH C_18_ column (Waters, 2.1 mm internal diameter × 50 mm length, and 1.7 µm particle size). Mass spectrometry (MS) analyses were performed using a QTof-Micro mass spectrometer (Waters, Milford, MA, USA). The parameters and conditions of ultra-high performance liquid chromatography (UHPLC)-MS analysis were set according to our previous study^47^. The mobile phases consisted of water with 0.05% (v/v) acetic acid (A) and methanol (B) delivered at 0.3 mL min^−1^. The elution started at a composition of 80% A and 20% B, held for 0.8 min; a 1.4 min linear gradient to 40% A, held for 3 min; return to the initial ratio of A and B by a 0.1 min gradient, held for 2.9 min. Detection conditions were the following: a column temperature of 25 °C; a capillary temperature of 300 °C; a spray voltage of 3000 V; an auxiliary pressure of 15 V; and a sheath pressure of 35 V.

### ABA extraction and determination

Extraction and determination of ABA were performed as described by Ré et al.^48^ with some modifications. One-half gram of ground lyophilized berries was extracted three times in 5 mL of cold 80% methanol (v/v) mixed with 30 μg mL^−1^ sodium diethyldithiocarbamate. The extracts were centrifuged at 8000 × *g* for 10 min, and the supernatant was concentrated to dryness under vacuum. The residue was dissolved in 4 ml of 0.4 M phosphate buffer (pH 8.0). After centrifuging, the aqueous phase was collected and supplemented with polyvinylpyrrolidone to remove the phenolics and then centrifuged at 8000 × *g* for 10 min. The supernatant was extracted with 4 mL of ethyl acetate (pH 3.0) twice. The upper phase was collected and concentrated to dryness under vacuum. The residue was dissolved in 1 mL of 0.5% (v/v) acetic acid/methanol (55:45, v/v) and finally filtered through a 0.45 μm filter.

ABA was quantified using an LC-electrospray ionization (ESI)-MS instrument. Ten microliters of the sample was injected into a Thermo Scientific Hypersil Gold column (50 × 2.1 mm, 1.9 μm) in a Scientific Ultimate 3000 HPLC system (Thermo, San Jose, CA, USA). The HPLC solvents were as follows: A, 0.04% acetate acid in water; and B, methanol (0.4 ml min^−1^). The two mobile phases were used in the gradient mode under the following time/concentration (min/%) of B: 0.0/20, 0.5/20, 2.5/90, 3.5/90, 3.6/20, and 5.0/20. Detection and quantification were performed using a TSQ Quantum Access MAX system (Thermo, San Jose, CA, USA). ABA was detected in the ESI negative mode and selected reaction monitoring with the following parameters: parent mass by charge (*m*/*z*) of 263.1; daughter mass by charge (*m*/*z*) of 153.0; collision energy of 14 eV.

### Determination of ethylene production rate

The ethylene production rate was measured using a GC-9A gas chromatograph (Shimadzu, Japan) equipped with a GDX-502 column and a flame ionization detector. The detached berries collected from different vines or the in vitro-treated berries were enclosed in a 2500-mL jar and incubated for 3 h. Five milliliters of the headspace gas was withdrawn from each jar through the septum stopper using a gas-tight syringe and was assayed.

### H_2_O_2_ extraction and determination

H_2_O_2_ was extracted and determined according to the methods of Patterson^49^ and Macnevin and Urone^50^. One gram of fresh berries was homogenized with 2.5 mL of chilled acetone and then centrifuged at 10 000 rpm for 15 min at 4 °C. One hundred microliters of the supernatant was incubated with 100 µL of Ti(SO_4_)_2_ (5%) and 200 µL of NH_3_·H_2_O. Centrifuged at 3000 rpm for 10 min, the precipitate was dissolved with 500 µL of 2 mol L^−1^ H_2_SO_4_, and the extract was immediately photometrically determined at 415 nm.

### Real-time quantitative reverse transcription-PCR

Total RNA was extracted using the hot borate method as described previously^51^. Two micrograms of total RNA was used to synthesize first-strand cDNA. The quantitative reverse transcription-PCR assays were conducted using a BIO-RAD iQ5 instrument (USA). All reactions were conducted in a 20-µL system containing 2 µL of Power SYBR Green PCR Master Mix (Applied Biosystems, Foster, USA), 3 µL of diluted cDNA, and 0.5 µM each primer. Fluorescence was measured at the end of each annealing step. The specificity of the amplification was determined with a dissociation curve analysis. Expression values were normalized against the *VvUbiquitin* gene. All primers are listed in Supplementary Table S1.

### Statistical analysis

The statistical analysis was performed by the SPSS (V19.0) statistical software package. A one-way analysis of variance followed by Duncan’s multiple range test was employed.

### Data availability

Data supporting the results can be found in this paper.

## Electronic supplementary material

Table S1. Sequences of the primers used in this paper

Fig. S1 Daily average temperature in the experimental site (36º.17'N, 117º.16'E) in 2016 and 2017
