# Supplementary material for: Melatonin promotes ripening of grape berry via increasing the levels of ABA, H2O2, and particularly ethylene
Source: Hortic Res. 2018 Aug 1;5:41. doi: 10.1038/s41438-018-0045-y (PMC6068098; doi:10.1038/s41438-018-0045-y)
Supplement: Supplementary file 1 — Table S1. Sequences of the primers used in this paper [file 41438_2018_45_MOESM1_ESM.docx]

**Table S1.** Sequences of the primers used in this paper

| Gene | Accession  Number | Forward primer (5'-3') | Reverse primer (5'-3') |
| --- | --- | --- | --- |
| *MYBA1* | AB097923 | GAGGGTGATTTTCCATTTGAT | CAAGAACAACTTTTGAACTTAAACAT |
| *UFGT* | AF000372 | GGGATGGTAATGGCTGTGG | ACATGGGTGGAGAGTGAGTT |
| *Ubiquitin1* | BN000705 | GTGGTATTATTGAGCCATCCTT | AACCTCCAATCCAGTTATCTAC |
