# Supplementary material for: Melatonin promotes ripening of grape berry via increasing the levels of ABA, H2O2, and particularly ethylene
Source: Hortic Res. 2018 Aug 1;5:41. doi: 10.1038/s41438-018-0045-y (PMC6068098; doi:10.1038/s41438-018-0045-y)
Supplement: Supplementary file 2 — Fig. S1 Daily average temperature in the experimental site (36º.17'N, 117º.16'E) in 2016 and 2017 [file 41438_2018_45_MOESM2_ESM.docx]

**Fig. S1 Daily average temperature in the experimental site (36º.17'N, 117º.16'E) in 2016 and 2017**
